# β_3_‐Adrenoceptor Agonist Effects on the Urinary Bladder Beyond Detrusor Relaxation

**DOI:** 10.1002/nau.70043

**Published:** 2025-03-31

**Authors:** Martin C. Michel, Ebru Arioglu‐Inan, Martin Hennenberg

**Affiliations:** ^1^ Dept. of Pharmacology University Medical Center, Johannes Gutenberg University Mainz Germany; ^2^ Dept. of Pharmacology, Faculty of Pharmacy Ankara University Ankara Türkiye; ^3^ Dept. of Urology LMU University Hospital, LMU Munich Munich Germany

**Keywords:** β_3_‐adrenoceptor, desensitization, fibrosis, hypertrophy, long‐term studies, mirabegron, vibegron

## Abstract

**Aims:**

β_3_‐Adrenoceptor agonists such as mirabegron or vibegron reach pharmacokinetic steady‐state within a few days. However, maximum improvements in symptoms of the overactive bladder syndrome are reached at time points later than 4 weeks, that is, detrusor smooth muscle relaxation cannot fully explain clinical effects. Therefore, we review mechanistic studies that have administered such drugs for longer periods of time.

**Methods:**

Narrative review.

**Results:**

The limited data indicates that β_3_‐adrenoceptor agonists reduce bladder fibrosis and hypertrophy and may induce cell proliferation. On the other hand, limited data also indicate that detrusor relaxation responses can partly desensitize with prolonged agonist exposure, possibly off‐setting effects on fibrosis and hypertrophy.

**Conclusions:**

Further long‐term studies with β_3_‐adrenoceptor agonists are needed to clarify the role of such processes in clinical improvement of patients with overactive bladder syndrome occurring at time points later than 4 weeks after initiation of treatment.

## 1 Introduction

β‐Adrenoceptors (β‐AR) are the main physiological mechanism to cause detrusor smooth muscle relaxation, thereby enabling bladder filling at low pressure during the storage phase of the micturition cycle [1]. While multiple subtypes of β‐AR are involved in this response in various mammalian species, β_3_‐AR are the main if not sole mediator in the human urinary bladder [2]. Mirabegron [3] and vibegron [4] are approved β_3_‐AR agonists for the treatment of the overactive bladder syndrome (OAB) and other agonists such as solabegron have also shown efficacy in the treatment of OAB [5]. Their overall clinical profile has been reviewed recently [6]. While β_3_‐AR‐mediated detrusor relaxation has been demonstrated in numerous studies [2], other possible cellular tissue targets have been proposed that may indirectly contribute to detrusor relaxation; these include the urothelium, afferent nerves, interstitial cells of Cajal, the major pelvic ganglion, blood vessels supplying bladder perfusion, and perhaps sites in the spinal cord and the brain [7]. The relative roles of these mechanisms remains to be determined.

β_3_‐AR agonists effectively relieve OAB symptoms but do not cure the condition, implying a need for chronic treatment [6]. The above mechanisms can potentially explain acute effects of β_3_‐AR agonists, that is, those occurring in the few days until a pharmacokinetic steady‐state has occurred. In contrast, they do not easily explain why it typically required more than 4 weeks of treatment before maximum improvement in OAB symptoms was observed in clinical trials [6]. This difference indicates that mechanisms beyond detrusor relaxation, be it by direct effects on smooth muscle or by indirect effects, are likely to exist. However, most experimental studies in the field have been based on exposure to a β_3_‐AR agonist for minutes to a few hours. Therefore, we will discuss here the limited number of experimental studies administering a β_3_‐AR agonist for longer than it takes to reach full exposure to the drug and examining mechanisms other than acute direct and indirect effects on detrusor smooth muscle tone.

## 2 Bladder Fibrosis

Fibrosis of the urinary bladder can occur in multiple conditions; irrespective of its cause, fibrosis can impair bladder compliance and thereby cause storage symptoms [8, 9]. One group of investigators induced endothelial injury in the iliac arteries, an established model to induce storage symptoms [10], by applying balloon injury plus a 2% cholesterol diet for 8 weeks with some rats additionally being treated with mirabegron (10 mg/kg/day orally) [11]. The endothelial damage was confirmed histologically but was not attenuated by treatment with mirabegron, indicating that mirabegron did not act on the vascular injury. Neither the endothelial injury nor the additional exposure to mirabegron affected body or bladder weight. However, the endothelial injury induced bladder dysfunction as indicated by a shortened micturition interval (from 10.74 to 5.49 min) and a reduced bladder capacity (from 1.79 to 0.91 mL), micturition volume (from 1.77 to 0.88 ml) and bladder compliance (from 0.15 to 0.09 mL/cm H_2_O), demonstrating that urine storage had been impaired as it also occurs in OAB patients. Concomitant treatment with mirabegron attenuated these impairments by about half. At the end of the 8 weeks of treatment, bladder tissue was excised to perform mechanistic in vitro experiments. Organ bath experiments found impaired detrusor contractions in response to KCl, the muscarinic receptor agonist carbachol, and to electric field stimulation that were not observed in tissue from rats concomitantly treated with mirabegron. The endothelial injury increased the collagen content in the muscle layer of the bladder wall (from 8.7% to 28.6%), which was attenuated by treatment with mirabegron (17.2%). This data provided the first evidence that chronic exposure to a β_3_‐AR agonist can attenuate the impairment of urine storage caused by a chronically reduced perfusion independent of alterations of perfusion and that mirabegron can partly or fully reduce the development of fibrosis in the urinary bladder.

## 3 Hypertrophy, Cell Proliferation and Programmed Cell Death

Bladder hypertrophy occurs under various pathophysiological conditions including BOO [12], bladder denervation [13], all models of type 1 diabetes [14], many but not all models of type 2 diabetes [15, 16], and in patients with OAB [17]. Irrespective of the cause of bladder hypertrophy, it is considered to be a pathophysiological factor leading to storage symptoms and overall bladder dysfunction [18, 19].

Bladder hypertrophy requires bladder smooth muscle proliferation and/or hyperplasia, sometimes accompanied by forms of programmed cell death [20]. Some investigators have exposed cultured human bladder smooth muscle cells to increased hydrostatic pressure and explored the effects of β‐AR agonists [21]. Exposure to increased hydrostatic pressure (100 cm H_2_O) increased the expression of the contractile marker α‐smooth muscle actin within 6 h at the mRNA and the protein level. The percentage of cells staining positive for proliferation marker 5‐ethynyl‐2′‐deoxyuridine (EdU) was also increased by increased hydrostatic pressure. Moreover, increased hydrostatic pressure down‐regulated the expression of β_2_‐AR and β_3_‐AR but did not affect that of β_1_‐AR. This pattern of regulation was also observed in a rat BOO study in this report. However, the latter effect may not be robust because BOO in our hands was associated with a decreased expression of β_1_‐AR, no change of β_2_‐AR, and an inconclusive change of β_3_‐AR mRNA [22]. The antagonists metoprolol (moderately selective for β_1_‐AR), ICI 118 551 (selective for β_2_‐AR) and SR 59 230 (one of the few antagonists potently inhibiting β_3_‐AR but not being selective for this subtype) did not affect the increase in α‐smooth muscle actin. Among β‐AR agonists, dobutamine (moderately selective for β_1_‐AR) did not affect the hydrostatic pressure‐induced increase in α‐smooth muscle actin, whereas formoterol (selective for β_2_‐AR) and BRL 37 344 (activating β_2_‐and β_3_‐AR) prevented the increase. This data provides evidence that activation of β_2_‐AR can impair a contractile phenotype induced by hydrostatic pressure; it also is compatible with the idea that β_3_‐AR agonists could mimic this, but is not conclusive in this regard due to limited selectivity of the applied tools.

Finally, preliminary data from our group were obtained in 10 male β_3_‐AR knock‐out in comparison to 12 wild‐type mice [23]. The β_3_‐AR knock‐out mice exhibited a markedly increased bladder weight (increase by 5.62 mg) but also an increased body weight (by 3.29 g); the bladder/body weight ratio was increased by 0.1 mg/g). These data are compatible with the idea that endogenous β_3_‐AR exhibit an anti‐hypertrophic effect on the urinary bladder. Another study from our group found that mirabegron induced increases in viability and of the proliferation marker Ki‐67 in human bladder smooth muscle cells, as well as downregulation of contractile proteins and M_2_ muscarinic receptors, within 40 h [24]. Additional studies with greater sample sizes in the knock‐out mice and studies in the treatment of established bladder hypertrophy in the context of a rat model of type 1 diabetes, and with prolonged exposure beyond 40 h in human bladder smooth muscle cells are currently ongoing.

Taken together, the current evidence points to an anti‐hypertrophic role of β_3_‐AR agonists in the urinary bladder but is not yet conclusive.

## 4 β_3_‐Adrenoceptor Desensitization

Muscarinic receptor antagonists inherently can only act on the OAB symptoms caused by acetylcholine, whereas β_3_‐AR agonists can also inhibit the detrusor contraction caused by other stimuli that may play a role under pathophysiological conditions [25]. Therefore, it had been hope that β_3_‐AR agonists could be more effective than muscarinic antagonists in the treatment of OAB, but clinical trials have not found major superiority in efficacy [6].

G protein‐coupled receptors including β‐AR typically undergo desensitization upon prolonged agonist exposure. Such desensitization can become limiting for treatment efficacy as found for instance for the use of β_2_‐AR agonists in the treatment of premature labor [26]. It is generally observed that β_3_‐AR are less sensitive to agonist‐induced desensitization as compared to β_1_‐ and β_2_‐AR, but several examples of agonist‐induced β_3_‐AR desensitization have been reported [27]. Thus, it is possible that agonist‐induced desensitization may limit the effectiveness of treatment with β_3_‐AR agonists. Such desensitization occurs within hours to days in models where it is observed [27], whereas the earliest time point in clinical OAB trials with β_3_‐AR agonists has been 2 weeks [6]. Thus, the available clinical data do not allow conclusions on the possibility of desensitization as a factor limiting the clinical effectiveness of β_3_‐AR agonists.

To our knowledge, only a single study has explored agonist‐induced desensitization of β‐AR‐mediated detrusor relaxation [28]. This study has used isolated detrusor strips from rat bladder exposed for 6 h to various β‐AR agonists. There after, the potentially desensitizing stimulus washed out and desensitization was assessed by relaxation responses to freshly added agonists. In contrast to the human bladder, the relaxation of rat detrusor is mediated by a combination of β_2_‐ and β_3_‐AR [2]. The study on rat detrusor strips found that desensitization was induced by isoprenaline (activating all β‐AR subtypes) and by fenoterol (selective for β_2_‐AR). Data on CL 316 243 (selective for β_3_‐AR in rodents) and mirabegron were ambiguous: based on the sample size in the study, they were neither different from placebo nor from isoprenaline in descriptive statistical analysis with a limited sample size. In vivo rat studies on the desensitization of detrusor relaxation upon long‐term treatment with mirabegron with greater statistical power are currently ongoing in our laboratories.

## 5 Conclusions and Perspectives

β_3_‐AR agonists acutely cause detrusor relaxation by direct and/or indirect mechanisms. However, the maximum improvement of storage symptoms in OAB patients typically occurs more than 4 weeks after initiation of treatment despite pharmacokinetic steady‐state occurring within a few days. Thus, we consider it likely that additional mechanisms beyond detrusor relaxation exist and are involved in the long‐term improvement of storage function.

Unfortunately, only few investigators have mechanistically studied the effects of prolonged β_3_‐AR agonist exposure. This limited number of studies indicates that β_3_‐AR agonists may have anti‐fibrotic and anti‐hypertrophic effects in the bladder, which could contribute to the improvement of storage function after the first 4 weeks of treatment. However, based on very limited data, the possibility exists that agonist‐induced desensitization of β_3_‐AR may limit the therapeutic effectiveness. We strongly recommend that considerably more experimental studies are conducted with long‐term (at least 1 week) treatment with β_3_‐AR agonists. The design of such studies should be analogous to clinical real world conditions, that is, with β_3_‐AR agonist treatment starting at a time point when pathology has already been established, and including prolonged exposure in cell culture studies.

The studies on effects of prolonged exposure to β_3_‐AR agonists were largely based on mirabegron. However, mirabegron can have effects unrelated to agonism at β_3_‐AR including antagonism at α_1_‐AR and binding to muscarinic receptors [29, 30, 31, 32] and indirect sympathomimetic effects [33]. Thus, other β_3_‐AR agonists should also be studied to obtain insight into whether the above are indeed effects of prolonged β_3_‐AR stimulation or specific for mirabegron and mediated by its off‐target effects.

A better understanding of the long‐term effects of β_3_‐AR agonists, particularly in a real treatment setting, may inform strategies to improve their clinical effectiveness.

## Author Contributions

Martin C. Michel, Ebru Arioglu‐Inan and Martin Hennenberg jointly conceived the manuscript and searched the literature. Martin C. Michel has drafted the manuscript. All authors have read the draft for critical intellectual content and approved the final version.

## Ethics Statement

The authors have nothing to report.

## Consent

The authors have nothing to report.

## Conflicts of Interest

M.C.M. has received consultancy and/or speaker honoraria related to β_3_‐AR from Astellas and Pierre Fabre. The other authors declare no conflicts of interest.

## Data Availability

The authors have nothing to report.
